# Management of Gastrointestinal Symptoms in Parkinson’s Disease

**DOI:** 10.1177/11795735251370014

**Published:** 2025-08-13

**Authors:** Han-Lin Chiang, Chin-Hsien Lin

**Affiliations:** 1 Division of General Neurology, Department of Neurology, Neurological Institute, 46615 Taipei Veterans General Hospital, Taipei, Taiwan; 2 School of Medicine, College of Medicine, National Yang Ming Chiao Tung University, Taipei, Taiwan; 3 Department of Neurology, 38006 National Taiwan University Hospital, College of Medicine, National Taiwan University, Taipei, Taiwan; 4 Graduate Institute of Biomedical Engineering, National Taiwan University, Taipei, Taiwan; 5 Graduate Institute of Molecular Medicine, College of Medicine, National Taiwan University, Taipei, Taiwan; 6 Graduate Institute of Brain and Mind Sciences, College of Medicine, National Taiwan University, Taipei, Taiwan

**Keywords:** Parkinson’s disease, gastrointestinal symptoms, gastroparesis, constipation, autonomic dysfunction, non-motor symptoms

## Abstract

Gastrointestinal (GI) dysfunction is a common and often underappreciated aspect of Parkinson’s disease (PD), with symptoms manifesting at multiple levels of the digestive tract, from swallowing difficulties to challenges with defecation. These non-motor symptoms can be more debilitating than the hallmark motor impairments of PD, profoundly affecting patients’ quality of life. The burden of GI issues in PD extends beyond discomfort, contributing to malnutrition, weight loss, and impaired medication absorption, which can exacerbate both motor and non-motor symptoms.

Despite their clinical significance, GI symptoms are frequently overlooked or mismanaged in routine practice. Inappropriate treatments, including certain medications and dietary recommendations, may inadvertently worsen the disease course. Therefore, a comprehensive understanding of GI dysfunction in PD is critical for clinicians, especially neurologists, to optimize patient care. This review provides an updated overview of the common GI manifestations in PD, including drooling, dyspepsia and dysphagia, gastroparesis, constipation, *H. pylori* infection, and small intestinal bacterial overgrowth. We discuss current diagnostic approaches, non-pharmacological and pharmacological treatment strategies. Recognizing and appropriately managing GI dysfunction in PD is essential for optimizing symptom control and improving patients’ overall well-being.

## Plain Language Summary

Many people with Parkinson’s disease (PD) experience digestive problems, but these issues are often overlooked. Symptoms can affect the entire digestive system, from difficulty swallowing to trouble with bowel movements. In some cases, these problems can be more challenging than the movement difficulties caused by PD. Poor digestion can lead to malnutrition, weight loss, and problems absorbing medication, which may worsen both motor and non-motor symptoms. Unfortunately, digestive issues in PD are sometimes mismanaged, and certain treatments or dietary changes might make things worse. It is important for doctors to understand and properly address these symptoms. This article provides an updated overview of common digestive problems in PD, including drooling, difficulty swallowing, slow stomach emptying, constipation, and infections in the gut. It also discusses how these issues can be diagnosed and treated using both medical and non-medical approaches. Proper management of digestive problems can help improve the quality of life for people with PD.

## Introduction

**Figure 1.**
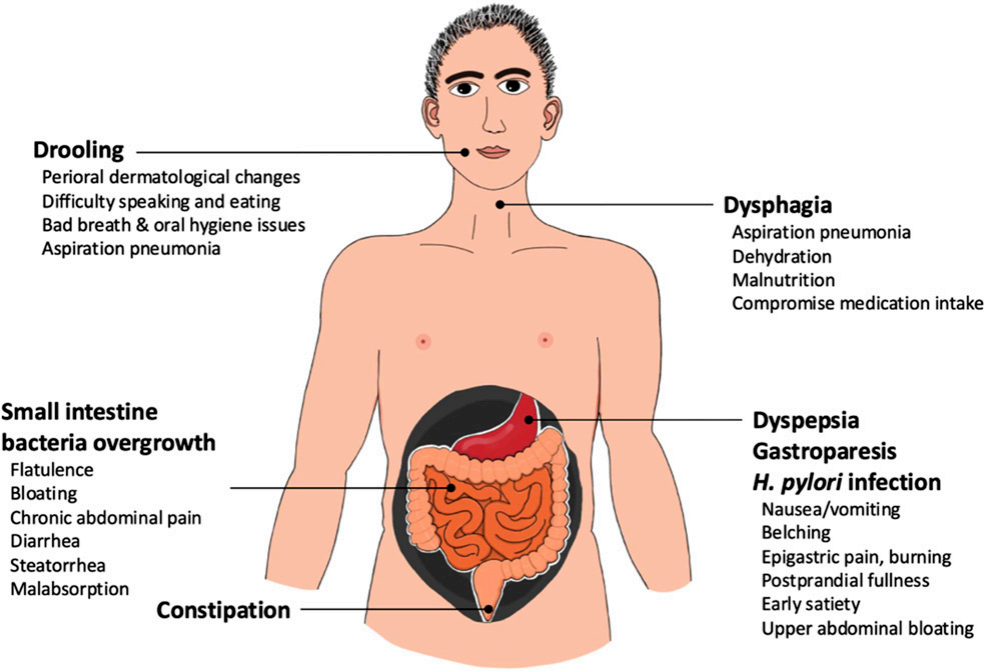
Common Gastrointestinal Dysfunctions and Associated Symptoms in Parkinson’s Disease. (The Drawing in This figure was Created by the First Author, Dr Han-Lin Chiang.)

Gastrointestinal (GI) dysfunction is a prevalent non-motor symptom of Parkinson’s disease (PD), affecting approximately 60%–80% of patients and contributing significantly to morbidity and reduced quality of life.^1^ In a large cross-sectional study involving 23 058 patients with PD, 81% of participants reported GI symptoms, second only to sleep disorders (96%), with constipation being the most prevalent GI symptom (64%).^2^ GI manifestations in PD span the entire digestive tract, presenting as impaired swallowing (dysphagia), delayed gastric emptying (gastroparesis), small intestinal dysmotility, constipation, and defecatory disorders (Figure 1). Recently, increasing attention has been garnered to GI symptoms manifesting not only after a PD diagnosis, but in fact, occurring before the occurrence of motor dysfunction. Constipation is the most common prodromal gastrointestinal symptom, affecting 50%–80% of patients.^3^ Other commonly reported GI symptoms in early stage of PD include dysphagia, dry mouth, nausea, and vomiting.^4,5^ Notably, constipation may precede motor symptoms by up to 2 decades, highlighting its potential role as an early clinical marker.^3^ Accordingly, constipation has been incorporated into the research diagnostic criteria for prodromal PD by the Movement Disorder Society Task Force on the definition of Parkinson’s Disease.^6^

The GI tract is one of the largest interfaces between the human body and the external environment. Often referred to as the “second brain,” the gut houses an extensive network of neurons within the enteric nervous system (ENS), with a neuronal count comparable to that of the spinal cord.^7^ This intricate gut neural network extends beyond enteric neurons and includes enteric glia, neurons of peripheral ganglia innervating the gut, intrinsic neurons, and specialized innervated epithelial sensors, such as enteroendocrine cells.^8^ The ENS is organized into 2 primary systems: the intrinsic and extrinsic components. The intrinsic ENS consists of the myenteric and submucosal plexuses, which regulate gut motility, secretion, and blood flow. The extrinsic innervation originates from the central nervous system (CNS) and connects to the gut through sympathetic and parasympathetic pathways, facilitating communication between the gut and the brain.^8^ The bidirectional communication between the ENS and CNS, termed the “gut-brain axis,” involves complex interactions among the neuroendocrine and neuroimmune systems. Key components of this axis include the hypothalamic–pituitary–adrenal axis, the autonomic nervous system (comprising both sympathetic and parasympathetic branches, including the ENS and the vagus nerve), and the gut microbiota residing within the gut lumen.^9^ Alterations in the gut microbiota have been proven to play a significant role in the pathogenesis of PD via the gut–brain axis, which encompasses interactions among the intestinal microbiota, microbial metabolites, mucosal immunity, and the vagus nerve in modulating brain function. Recent multi-cohort integrative analyses and meta-analyses have identified a PD-associated microbial profile enriched in pro-inflammatory species. These dysbiotic changes are linked to compromised intestinal barrier integrity (“leaky gut”), systemic inflammation, and altered microbial metabolic functions and gene expression profiles.^10,11^ Growing evidence supports the involvement of the GI tract and the ENS in the pathophysiology of PD. Key pathological findings include the aggregation of misfolded α-synuclein within enteric neurons, intestinal inflammation, and compromised gut barrier integrity.^12,13^ These alterations may contribute to the initiation and progression of PD, highlighting the potential of the gut as a critical site of early disease processes.

The etiology of GI dysfunction in PD is multifactorial, involving neurodegenerative, autonomic, and peripheral mechanisms. A critical pathological hallmark of PD is the aggregation of misfolded α-synuclein into Lewy bodies, which occurs not only in the CNS but also in the ENS.^14^ Braak’s hypothesis postulates that α-synuclein pathology may originate in the gut and ascend via the vagus nerve to the brainstem, suggesting a gut-to-brain axis in PD pathogenesis.^15^ In support of this, α-synuclein aggregates have been identified in the enteric neurons of the myenteric and submucosal plexuses, even before the onset of classic motor symptoms. The distribution of α-synuclein pathology may explain the early non-motor symptoms of PD, particularly constipation, which can precede motor symptoms by decades.^3^ Dysautonomia also plays a critical role in GI dysfunction in PD. The vagus nerve, a key component of the parasympathetic nervous system, modulates GI motility and secretion. Vagal degeneration and autonomic failure lead to impaired GI motility, contributing to symptoms such as gastroparesis and constipation.^12,13^ Furthermore, enteric neuron degeneration disrupts the complex network of excitatory and inhibitory signals required for coordinated gut peristalsis. In addition to neuronal factors, alterations in the gut microbiota (dysbiosis) have been implicated in PD. Dysbiosis may influence GI motility and permeability through the production of microbial metabolites, which could promote α-synuclein misfolding and propagation. Increased intestinal permeability (“leaky gut”) might also facilitate systemic inflammation and impact the ENS and CNS.^12,13^

The clinical burden of GI dysfunction spans the entire course of PD, from the prodromal phase, where symptoms such as constipation may serve as early indicators, to the advanced stage, where GI disturbances can interfere with the absorption of antiparkinsonian medications and worsen motor fluctuations. Despite its prevalence, the management of GI symptoms in PD remains challenging due to the limited availability of pharmacological treatments supported by robust evidence, and the underutilization of non-pharmacological approaches. This review aims to provide a comprehensive and updated overview of common GI dysfunctions in PD, including drooling, dysphagia, dyspepsia and gastroparesis, constipation, *Helicobacter pylori* infection, and small intestinal bacterial overgrowth (SIBO). Each section discusses the clinical features, underlying pathophysiology, diagnostic approaches, and available treatment options. Through this structure, we aim to offer clinicians practical guidance to improve the recognition and management of GI symptoms in PD

## Methodology

We performed a literature search using PubMed covering the period from 1969 to March 1, 2024. Search terms included “Parkinson’s disease,” “parkinsonism,” “gastrointestinal,” “drooling,” “dysphagia,” “dyspepsia,” “gastroparesis,” “constipation,” “Helicobacter pylori,” “small intestinal bacterial overgrowth,” and “non-motor symptoms,” with the language restricted to English. References were selected based on their relevance to the topic, scientific rigor, recency, and inclusion of comprehensive or high-impact findings. Priority was given to original studies and up-to-date reviews directly addressing gastrointestinal symptoms in PD.

## Drooling

### Epidemiology and Clinical Manifestation

Drooling, also known as sialorrhea, is the excessive buildup of saliva in the mouth, often leading to unintentional spillage. It is common in PD and can appear early in the disease course. The reported prevalence ranges widely from 10% to 84% due to the lack of standardized diagnostic criteria and the use of different screening tools.^16^ The adverse effects of drooling are multifaceted, encompassing physical and psychosocial challenges, and ultimately reduce quality of life.^17,18^ Physically, drooling can cause perioral dermatological changes, including chapping, excoriation, dermatitis, difficulty speaking and eating, bad breath, and oral hygiene issues due to stagnant saliva and bacterial overgrowth.^16,17,19^ Saliva pooling in the throat can lead to coughing and aspiration pneumonia.^16,19^ Psychosocially, drooling leads to embarrassment, social withdrawal, and reduced physical contact, increasing isolation in PD.^16^ Caregivers also face added burdens, including frequent laundry, hygiene management, and restricted social activities.^19^ In a survey of 265 PD patients, it was ranked among the 24 most bothersome symptoms—15^th^ in those with up to 6 years of disease and third in those with more than 6 years.^20^ Despite its significant impact on patients and caregivers, it remains underrecognized by physicians and underreported by patients, ultimately leading to undertreatment.^19^

### Diagnosis

The most commonly used tool for screening drooling in patients with PD is the Sialorrhea Clinical Scale for Parkinson’s Disease (SCS-PD).^21^ Other tools used in clinical settings include the Non-Motor Symptoms Questionnaire (NMSQuest),^22^ which includes a single item on drooling and is useful for broader non-motor symptom screening.

### Pathophysiology and Treatment

Previous studies have suggested that drooling in PD is unlikely to be related to increased saliva production^23^ but rather to dysfunctional orolingual and pharyngeal motor control, infrequent swallowing,^24^ severe hypomimia, unintentional mouth opening, and stooped posture.^25^ Therefore, in addition to pharmacological therapy, which primarily aims to reduce saliva production, non-pharmacological interventions such as behavioral modification and speech therapy are also available. More invasive treatments, including radiation therapy and surgical interventions, are considered as a last resort when both non-pharmacological and pharmacological approaches fail.^26^ Due to the limited evidence from large randomized controlled trials (RCTs) on the treatment of drooling in PD, current treatment options, with the exception of botulinum toxin injections, are primarily based on expert opinions or relatively small-scale studies.

#### Non-Pharmacological Therapy

For mild symptoms, drooling can be managed by using sugar-free candy or gum in social situations to promote more frequent swallowing.^27^ Postural techniques, such as using a wheelchair with the head tilted back, can be recommended to the patients.^28^ Various prosthetic devices, such as chin cups and dental appliances, can potentially help patients by improving mandibular stability, lip closure, tongue positioning, and swallowing.^29^ Speech pathologists use behavioral and swallowing therapy, including cues, oral-facial techniques, and exercises, to improve oral motor control, jaw stability, tongue movement, and lip closure, reducing nasal regurgitation and enhancing saliva management and swallowing safety.^29^ However, the effectiveness of most of the non-pharmacological therapies described above for managing drooling in PD remains unproven in clinical trials. Marks et al. treated 6 PD patients with swallowing behavior therapy, including saliva control education, a drooling awareness chart, and a swallow reminder brooch that beeped at regular intervals.^30^ The intervention reduced drooling severity on the authors’ drooling rating scale, though the effect waned by 3 months.^30^ A single-arm study evaluated the effects of 6 to 8 weeks of expiratory muscle strength training (using a handheld device requiring patients to exhale against a threshold load) in 16 PD patients to enhance swallowing efficiency and lip closure. The study showed improvements in the SCS-PD,^21^ swallowing function (assessed by the Mann assessment of swallowing ability)^31^ and the Swallowing Quality of Life Questionnaire,^32^ and peak cough flow, with no reported adverse effects.^33^

#### Pharmacological Therapies

The UK National Institute for Health and Care Excellence (NICE) guideline recommends considering pharmacological treatment only if non-pharmacological therapy has failed or is unavailable for patients with PD-related drooling.^34^ Because drooling often occurs during medication “off” periods,^16^ optimizing dopaminergic therapy to improve swallowing function can be a reasonable initial strategy for treatment. Pharmacological therapies for drooling in PD include botulinum toxin injections and oral medications.

The major salivary glands produce approximately 1.5 liters of saliva per day, with the parotid gland contributing 25% of total secretion, primarily in response to olfactory and gustatory stimuli, while the submandibular gland and sublingual gland account for 70% and 5% of salivary output, respectively.^35^ Botulinum toxin injections targeting the parotid and submandibular glands to inhibit acetylcholine release from postganglionic parasympathetic nerve endings^36^ have gained substantial evidence over the years demonstrating efficacy. The results of the RCTs on botulinum toxin injections for treating PD-related drooling are summarized in Table 1. To date, 2 large-scale studies have investigated the effect of botulinum toxin injections on PD-drooling. The SIAXI study is a Phase III trial evaluating the efficacy and safety of incobotulinumtoxinA in patients with neurological disorders (n = 184), the majority of whom had PD (70.7%).^41^ The study demonstrated significant improvements in its co-primary endpoints in the 100U (30U per parotid gland, 20U per submandibular gland) group compared to placebo, including a reduction in unstimulated salivary flow rate (uSFR, objective measure) (*P* = 0.004) and improved Patient’s Global Impression of Change Scale scores (GICS) (*P* = 0.002). The most common treatment-related adverse effects were dry mouth (5.4% in the 75U group and 2.7% in the 100U group) and dysphagia (2.7% in the 75U group, none reported in the 100U group).^41^ In the extension phase of the SIAXI study, subjects continued for 3 additional injection cycles (each 16 ± 2 weeks) and maintained sustained benefit without an increase in adverse event incidence.^47^ The RIMA study evaluated the safety, efficacy, and tolerability of rimabotulinumtoxinB in 176 adults with troublesome sialorrhea, with patients with PD comprising 65.2% of the study population.^44^ In the study, both 2500U (1000U per parotid gland, 250U per submandibular gland) and 3500U (1500U per parotid gland, 250U per submandibular gland) significantly improved the primary endpoint of uSFR (*P* < 0.001) and Clinical Global Impression of Change (CGI-C) (*P* < 0.001) at week 4.^44^ The effect of the injections (uSFR and CGI-C) was observed as early as one week with both doses and sustained through week 8. The most common side effects, all of which were self-limited, included dry mouth (38.1% in the 2500U group and 45.3% in the 3500U group), dysphagia (11.1% and 4.7%), and dental caries (7.9% and 4.7%), respectively.^44^ Consequently, the FDA has approved both incobotulinumtoxinA and rimabotulinumtoxinB for the treatment of chronic sialorrhea in adults.

**Table 1.** Summary of Randomized Controlled Trials on Botulinum Toxin Injection for Drooling Symptoms in Patients With PD

Study	Botulinum toxin	No. of patients	Diagnosis	Location (dose on each side, unit)	US/A	Outcome	Side effects
Botulinum toxin type A							
Mancini 2003^37^	AbobotulinumtoxinA	Placebo:10 BoNT:10	PD:14 MSA:6	PG (146.25) SMG (78.75)	US	DSFS significantly improved	None
Lipp 2003^38^	AbobotulinumtoxinA	Placebo:7 18.75U:8 37.5U:9 75U:8	PD:12 ALS:12 MSA:4 CBD:4	PG (18.75, 37.5, 75)	A	Significant decrease of saliva weight in the 75 unit group	None
Lagalla 2006^39^	OnabotulinumtoxinA	Placebo:16 BoNT:16	PD	PG (50)	A	Significant improvement of VAS-D, VAS-FD, VAS-SD, UPDRS-ADL subscore for drooling, saliva weight	Swallow:1
Narayanaswami 2016^40^	IncobotulinumtoxinA	Crossover design:9	PD	PG (20) SMG (30)	A	No difference in DSFS and saliva weight	Chewing, tongue control:1
Jost 2019^41^	IncobotulinumtoxinA	Placebo:32 75U:69 100U:72	PD:130 aPM:16 Stroke:33 TBI:5	PG (22.5, 30) SMG (15, 20)	A or US	Significant improvement in uSFR, GICS (primary endpoint)	Dry mouth: 5.4%(75U) 2.7%(100U) dysphagia: 2.7%(75U)
Botulinum toxin type B							
Ondo 2004^42^	RimabotulinumtoxinB	Placebo:8 BoNT:8	PD	PG (1000) SMG (250)	A	Significant improvement in VAS, DRS, DSFS	Dry mouth:3 Worsened gait:2 Diarrhea:1 Neck pain:1
Lagalla 2009^43^	RimabotulinumtoxinB	Placebo:18 BoNT:18	PD	PG (4000)	A	Significant improvement in DSFS, VAS-FD, VAS-SD, UPDRS-ADL subscore, CGI, saliva weight	Swallow:3
Chinnapongse 2011	RimabotulinumtoxinB	Placebo:15 BoNT 1500:14 2500:12 3500:13	PD	PG (500, 1000, 1500) SMG (250)	A	Significant improvement in DSFS in dose dependent manner and salivary flow rate	Dry mouth:1 Breath odor:1 Dysgeusia:1
Isaacson 2020^44^	RimabotulinumtoxinB	Placebo:57 2500:63 3500:64	PD:119 Stroke:13 ALS:12 CP:4 Medi: 6 Other:30	PG (1000) SMG (250)	A or US	Significant improvement in the uSFR change, CGI-C (primary endpoint)	Dry mouth: 38.1%(2500U) 45.3%(3500U) Dysphagia: 11.1%(2500U) 4.7%(3500U) Dental caries 7.9%(2500U) 4.7%(3500U)
Botulinum toxin type A & B							
Guidubaldi 2011^45^	AbobotulinumtoxinA RimabotulinumtoxinB	Crossover design: 14	PD:7 ALS:7	PG (A:100; B:1000) SMG (A:25; B:250)	US	Significant improvement in DSFS, DRS, VAS, CGI, and saliva weight	Dry mouth (26.8%) Viscous saliva (14.6%)

No., number; pts, patients; Dx, disease; BTX, botulinum toxin; PG, parotid gland; SMG, submandibular gland; A, localization using anatomical marker; US, localization under ultrasound guidance; PD, Parkinson’s disease; ALS, amyotrophic lateral sclerosis; MSA, multiple system atrophy; CBD, corticobasal degeneration; CP, cerebral palsy; Medi, medication induced; DSFS, Drooling Severity and Frequency scales^46^; VAS, visual analogue scale^37^; DRS, drooling rating scale^24^; VAS-D, visual analogue rating of drooling frequency; VAS-FD, VAS assessment of patient embarrassment within the familial context; VAS-SD, VAS assessment of patient embarrassment within the social context; UPDRS, unified Parkinson’s disease rating scale; CGI, clinical global impression; TBI, traumatic brain injury; uSFR, unstimulated flow rate; GICS, patient rated global impression of change scale; CGI-C, clinical global impression of change.

Only one study compared the difference between botulinum toxin type A and B in treating drooling. In a small randomized, double-blind, crossover pilot study, 14 patients with PD or amyotrophic lateral sclerosis were treated with 250U of abobotulinumtoxinA and 2500U of rimabotulimtoxinB. While the mean duration of benefit was similar between the 2 treatments, the onset latency was significantly shorter with rimabotulinumtoxinB (2.4 ± 1.4 days) compared to abobotulinumtoxinA (5.9 ± 3.3 days) (*P* = 0.001).^45^ Further head-to-head trials are needed to draw definite conclusions.

Salivary production is primarily regulated by the parasympathetic system through cholinergic activation of muscarinic M3 receptors, while the sympathetic system plays a modulatory role by influencing saliva composition rather than overall secretion.^48^ The results of clinical trials on oral or sublingual pharmacological therapy for drooling in PD are summarized in Table 2.^46,48-55^ Due to small-scale studies and limited clinical evidence, no oral medication has received FDA approval for the treatment of drooling. The use of anticholinergics to treat drooling in PD should be cautious due to their potential systemic and central nervous system side effects.^19^ Clinical trials for treating PD-drooling have targeted drugs administered locally (e.g., sublingual) or those with stronger muscarinic M3 activity that do not cross the blood-brain barriers (BBB) to enhance efficacy while minimizing systemic side effects. Glycopyrrolate is an anticholinergic with a quaternary ammonium structure that does not cross the BBB. To date, there are 2 RCTs investigating the efficacy of glycopyrrolate in treating PD-drooling.^50,52^ In a 4-week, randomized controlled crossover trial, glycopyrrolate (1 mg 3 times daily) was administered to 23 patients with PD. The treatment group showed a significantly higher responder rate (39.1%) compared to the placebo group (4.3%) (*P* = 0.021).^50^ A subsequent 12-week phase II randomized controlled trial evaluated glycopyrrolate (up to 4.5 mg) in 28 PD patients. Glycopyrrolate treatment significantly improved the Radboud Oral Motor Inventory for PD–Saliva score compared to placebo (between-group difference, 5.3; 95% confidence interval, 1.0-9.6), with dry mouth being the most common adverse effect.^53^ These studies provided supportive evidence of glycopyrrolate’s sustained efficacy in reducing drooling of PD patients.

**Table 2.** Summary of Oral or Sublingual Pharmacological Therapies for Drooling Symptoms in Patients With PD

Study	Drug	Study design	No. of pts	Outcome	Adverse effects
Anticholinergics					
Hyson 2002^46^	Atropine 0.5 mg 1% wt/vol solution sublingual 1 drop 2x/d, 1 wk	OL	6 PD, 1 PSP	Improvement in dental roll weight and subjective (a Likert-type drooling scale) measurements	Delirium:1, hallucination:2
Thomsen 2007^49^	Sublingual ipratropium bromide spray (21mcg/spray) 1-2 sprays, max. 4x/d, 2 wks	RCT, cross over	17 PD	No change in dental roll weight; improvement in UPDRS drooling subscore but post-hoc analysis was not significant	dry nasal passage and nose bleed: 1
Arbouw 2010^50^	Glycopyrrolate 1 mg 3x/d, 1 wk	RCT, cross over	23 PD	Significantly better responder rate and improvement in the mean sialorrhea score	Statistically insignificant
Lloret 2011^51^	3 doses of intra-oral tropicamide films 0.3 mg, 1 mg, 3 mg in random (separated by 7 days)	RCT	19 PD	No significant improvement in the visual analog scale (VAS) and saliva volume compared to placebo	Statistically insignificant
Mestre 2020^52^	Glycopyrrolate up to 4.5 mg, 12 wks	RCT	28 PD	Significant improvement in the ROMP–Saliva^53^	Dry mouth, constipation, worsening of cognition at higher dose
Adrenergic receptor agonists/antagonists					
Serrano-Dueñas 2003^54^	Clonidine 0.15 mg/d, 3 months	RCT	32 PD	Drooling frequency (wiping episodes per 5 min) and treatment response scale significantly improved	Statistically insignificant
Cheng 2019^55^	Dihydroergotoxine mesylate 2.5 mg 2x/d, 2 wks	OL + RCT, cross over	20 PD	Significantly better response rate and improvement in the SCS-PD score^25^	Statistically insignificant

Abbreviations: wks, weeks; d, day; OL, open label; RCT, randomized controlled trial, PD, Parkinson’s disease; PSP, progressive supranuclear palsy; UPDRS, Unified Parkinson’s Disease Rating Scale; ROMP-saliva, Radboud Oral Motor Inventory for Parkinson’s Disease–Saliva; SCS-PD, Sialorrhea Clinical Scale for Parkinson’s Disease.

The mechanisms by which adrenergic receptor agonists and antagonists affect salivary control remain less well understood. Two studies evaluated the effect of adrenergic receptor agonist/ or antagonist in treating PD-drooling. One double-blind, placebo-controlled trial included 32 patients, who were followed for 3 months, and showed that clonidine (0.15 mg/day), an α-2 adrenergic receptor agonist, significantly improved drooling frequency, as measured by the number of wiping episodes per 5 min.^54^ A study evaluated dihydroergotoxine mesylate (2.5 mg twice daily), a mixed α1/α2 adrenergic antagonist, in 2 phases.^55^ The first phase was a 3-week, open-label, single-arm trial involving 10 patients, which demonstrated significant improvement in the UPDRS sialorrhea subscore (*P* = 0.004) and SCS-PD (*P* = 0.005), with a response rate of 60%. The second phase was a 6-week, randomized, placebo-controlled crossover trial involving 20 patients, which showed significantly greater improvement in the UPDRS sialorrhea subscore, SCS-PD, and response rate in the treatment group (*P* = 0.001, *P* < 0.001, and *P* = 0.003, respectively).^45^ There were no significant side effects in both studies.

Finally, surgical intervention, including various procedures such as sublingual or submandibular gland excision, submandibular or parotid duct ligation, and submandibular or parotid duct rerouting, either individually or in combination, remains a last resort for patients with severe drooling. The role of surgical intervention has not been studied in patients with PD, and the different surgical techniques and their outcomes in drooling management are reviewed elsewhere.^56,57^

## Dysphagia

### Epidemiology and Clinical Manifestation

The prevalence of dysphagia in PD ranges from 11% to 87%, varying based on disease stage, duration, and assessment methods.^58,59,60^ Although dysphagia commonly worsens with disease progression and becomes more evident in the later stages of PD, early signs can emerge during the prodromal or early phases.^61^ Nevertheless, it often remains undiagnosed until more advanced stages, likely due to its subtle onset and under-recognition in early clinical evaluations.

The presence of dysphagia in patients with PD compromises medication intake and increases the risk of dehydration, malnutrition, and aspiration pneumonia.^62^ Certain clinical features may help clinicians identify dysphagia in PD. These include advanced disease stage, unexplained weight loss or a body mass index below 20, presence of drooling, and coexisting dementia.^24,62-65^ There is currently no pharmacological therapy that directly treats dysphagia in PD. Management often requires a multidisciplinary approach, involving specialists such as otorhinolaryngologists, gastroenterologists, speech-language pathologists, and dietitians to optimize care and address the various aspects of swallowing dysfunction.^66^

### Diagnosis

Videofluoroscopic evaluation remains the most reliable method for diagnosing oropharyngeal dysfunction in individuals with PD.^67^ The following clinical scales and questionnaires are commonly used to screen for dysphagia in people with PD: The Swallowing Disturbance Questionnaire (SDQ)^68^ is a self-reported tool comprising 15 items focused on swallowing difficulties. The Munich Dysphagia Test–Parkinson’s Disease (MDT-PD)^69^ is another self-administered questionnaire with 26 items across 4 categories, designed to detect early oropharyngeal symptoms and assess the risk of laryngeal penetration and/or aspiration. The Swallowing Clinical Assessment Score in Parkinson’s Disease (SCAS-PD)^70^ is a clinician-administered scale with 12 items, aimed at identifying impairments in the oral and pharyngeal phases of swallowing in PD.

### Pathophysiology and Treatment

Patients with PD and dysphagia can have impairments across all phases of swallowing. In the oral phase, common findings include repetitive pump-like tongue movements, oral residue, premature spillage, and piecemeal deglutition, reflecting impaired bolus control and propulsion. The pharyngeal phase is often characterized by residue accumulation in the valleculae and pyriform sinuses, aspiration, somatosensory deficits, and a reduced rate of spontaneous swallows. In the esophageal phase, hypomotility, spasms, and multiple contractions are observed.^62^ These abnormalities underscore the complex and multi-phase nature of dysphagia in PD; therefore, it is suggested that treatment be guided by instrumental findings (Table 3).^66^ Currently, key therapeutic directions for dysphagia in PD include oral care (to prevent aspiration pneumonia),^76^ optimization of PD treatments, swallowing therapy and botulinum toxin injection for upper esophageal sphincter (UES) dysfunction.^66^ A comprehensive review by Bhidayasiri et al. provides detailed insights into the management of dysphagia in PD in real-world clinical practice.^77^

**Table 3.** Objective Evaluations of Gastrointestinal Dysfunction

**Drooling** ^71^
Bib count
Bib weight
Sochaniwskyj’s technique
Drooling Quotient
5-min Drooling Quotient (DQ5)
**Dysphagia** ^66,72^
Bedside screening
Cough reflex testing
Modified barium swallow studies
Videofluoroscopy swallowing studies (VSS)
High resolution manometry
Fiberoptic endoscopic evaluation of swallowing (FEES)
Swallowing electromyography
**Gastroparesis** ^,72,73^
Gastric emptying scintigraphy (golden standard)
^13^C-sodium octanoate gastric emptying breath test (FDA-approved)
Wireless motility capsule (WMC) (FDA-approved*)
Electrogastrography
**Helicobacter pylori infection** ^74^
Urea breath test
Fecal antigen test
Serologic testing
Upper endoscopy with rapid urease test on the gastric biopsy tissue
**Small bowel bacteria overgrowth** ^74^
Breath tests (glucose or lactulose breath tests)
Small bowel aspiration with culture (golden standard)
**Constipation** ^72,75^
Radiopaque marker study for colonic transit
Wireless motility capsule (WMC)
Endoscopy
Anorectal manometry
Balloon expulsion testing
Barium enema
Defecography
Magnetic resonance defecography

*not endorsed by the United European Gastroenterology and European Society for Neurogastroenterology and Motility.

While studies have shown that dopaminergic medications can potentially improve dysphagia in PD, especially in the early stages,^78^ not all patients respond to dopaminergic treatment. In a study by Fuh et al, only half of the patients demonstrated objective improvement in swallowing function after levodopa administration.^79^ On the other hand, the opposite effects were observed.^80,81^ For patients whose swallowing function is responsive to dopaminergic therapy, the timing of taking the medication should be planned to ensure they are in their optimal “ON” state during mealtime, typically 30 to 60 min before eating.^66,82^

Swallowing therapy remains the mainstay treatment for dysphagia in PD, focusing on compensatory strategies, dietary modifications, and exercise-based interventions to improve swallowing function and reduce aspiration risk. However, swallowing therapy varies across different settings due to differences in clinical protocols, patient characteristics, and available resources. While most studies using swallowing therapy have demonstrated improvements in swallowing function,^83^ there is currently insufficient high-quality evidence to determine which protocol is most effective for improving dysphagia in PD. A detailed review of different swallowing therapies is beyond the scope of this review and is discussed elsewhere.^83,84^ Transcutaneous electrical stimulation (TES) activates nerves and motor end plates to promote muscle retraining, inducing functional muscle contractions as a supplement to swallowing therapy. However, studies have not shown significant additional benefits of TES for dysphagia in PD beyond conventional swallowing therapy.^66^ Non-invasive brain stimulation (NIBS), including repetitive transcranial magnetic stimulation (rTMS) and transcranial direct current stimulation (tDCS), has emerged as an adjunct therapy for dysphagia rehabilitation, particularly in patients with stroke. Only a few studies have investigated the effect of NIBS on patients with PD. In a randomized-controlled crossover study, the authors randomly assigned 40 patients with dysphagia, including 9 with PD, to either the tDCS or rTMS (intermittent theta-burst stimulation [iTBS]) group. Swallowing function was assessed using the Dysphagia Outcome and Severity Scale (DOSS).^85^ Both the tDCS and iTBS groups showed improvement in swallowing function at one month post-treatment (*P* = 0.014 and *P* = 0.001, respectively). However, only the iTBS group exhibited sustained improvement at 3 months post-treatment.^86^ Another randomized controlled study recruited 33 patients with advanced PD and randomized them in a 1:2 ratio to receive either sham or rTMS over the hand area of each motor cortex. The rTMS protocol consisted of 2000 pulses at 20 Hz, delivered at 90% of the resting motor threshold, with 10 trains of 10 s each, separated by 25-second intervals. Stimulation was applied for 5 min on each hemisphere for 10 days (5 days per week), followed by 5 booster sessions, administered once per month for 3 months. There was a significant and long-lasting (3 months) improvement in the Arabic Dysphagia Handicap Index,^87^ as well as in pharyngeal transit time and time to maximal hyoid elevation measured by videofluoroscopy, following rTMS treatment compared to the sham stimulation group.^88^ For patients whose dysphagia is associated with spasm or reduced relaxation of the UES, 2 single-arm studies by the same group suggest that unilateral botulinum toxin injection into the cricopharyngeal muscle, using incobotulinumtoxinA (15-20U)^89^ or onabotulinumtoxinA (15U),^90^ is beneficial for about half of the patients, including those with PD. Although no side effects were reported in the first study with 34 patients,^89^ 2 out of 67 patients in the second study developed severe aspiration after the second injection.^90^ Notably, both patients had also not responded to the first injection. Due to the small sample size of these trials, large-scale randomized controlled studies are needed to confirm their findings.

## Dyspepsia and Gastroparesis

### Epidemiology and Clinical Manifestation

Dyspepsia is a general term used to describe symptoms that arise from the gastroduodenal region. Dyspeptic symptoms include epigastric pain, burning, postprandial fullness, early satiety, and other issues such as upper abdominal bloating, nausea, vomiting, and belching.^91^ Dyspepsia can be caused by an organic disease or can be functional, with no identifiable structural explanation. No studies have determined the true prevalence of functional dyspepsia in PD. However, one study used the Leeds Dyspepsia Questionnaire^92^ to assess dyspeptic symptoms in 103 patients with PD and 81 healthy controls. They found that dyspeptic symptoms are more common in patients with PD (*P* = 0.001), with the 3 most troublesome symptoms, in order of reported frequency, being excessive fullness/bloating (20.4%), indigestion (18.4%), and nausea (15.6%).^93^

Gastroparesis is defined as delayed gastric emptying in the absence of mechanical obstruction. The true prevalence of gastroparesis remains uncertain due to the lack of standardized methodology and the limited scale of existing studies.^94^ However, a systematic review reported that delayed gastric emptying is highly prevalent among PD patients attending neurology clinics, with estimates ranging from 35% to 100%, though many cases may be asymptomatic.^95,96^ Besides causing discomfort with symptoms such as nausea and vomiting (cardinal symptoms), postprandial fullness, early satiation, epigastric pain, and bloating, gastroparesis also contributes to motor fluctuations by impairing the timely delivery of levodopa to the small intestine for absorption and breaking down of levodopa by the DOPA decarboxylase and catechol-O-methyltransferase in the gut mucosa.^97^ Gastroparesis symptoms are non-specific and overlap with other GI conditions, such as functional dyspepsia or rapid gastric emptying.^73,98,99^ In a study by Balan et al, among 572 patients clinically suspected of having delayed gastric emptying, only 165 (29%) were confirmed via gastric emptying scintigraphy. Given the low positive predictive value, diagnosis of gastroparesis should rely on objective measures rather than clinical symptoms alone.^62,86,87^ Gastric emptying scintigraphy is the gold standard for diagnosing gastroparesis, while ^13^C gastric emptying breath tests and wireless motility capsules, also FDA-approved, serve as alternative methods, though they are less accurate (Table 3).^86^

### Pathophysiology and Treatment

The pathophysiology of functional dyspepsia is complex and multifactorial. The putative pathophysiological mechanisms include impaired gastric emptying, decreased fundic accommodation, gastroesophageal reflux, *Helicobacter pylori* (*H. pylori*) infection, gastric or duodenal hypersensitivity to acid, distention, and other intraluminal stimuli, gastric or duodenal inflammation/infection, and psychosocial factors.^100^ The mainstay of treatment includes *H. pylori* eradication, proton pump inhibitors, prokinetics, and antidepressants, particularly tricyclic antidepressants.^91,100,101^ Importantly, many prokinetics are centrally acting dopamine antagonists and are contraindicated in patients with PD.

Two recent consensus statements on gastroparesis treatment have been published, highlighting dietary and pharmacological management options.^102,103^ Dietary strategies include avoiding high-fiber and high-fat foods, eating small, frequent meals, and incorporating liquid-based meals (e.g., soups) and small-particle-size diets, which are less affected by delayed gastric emptying.^104^ As for pharmacological therapy, it is important to recognize that medications used to manage motor symptoms in PD or other concomitant disease may also impact gastric motility. Studies have shown that levodopa and anticholinergics inhibit gastric emptying in healthy subjects.^105-108^ Studies on the effect of levodopa in patients with PD are conflicting. While some studies found that levodopa delays gastric emptying, others reported similar or faster gastric emptying compared to controls.^94,109-112^ Notably, medications or certain dietary component commonly used for other GI symptoms, such as proton pump inhibitors^113^ and soluble fiber,^114^ may exacerbate gastroparesis and should therefore be used with careful considerations.

**Figure 2.**
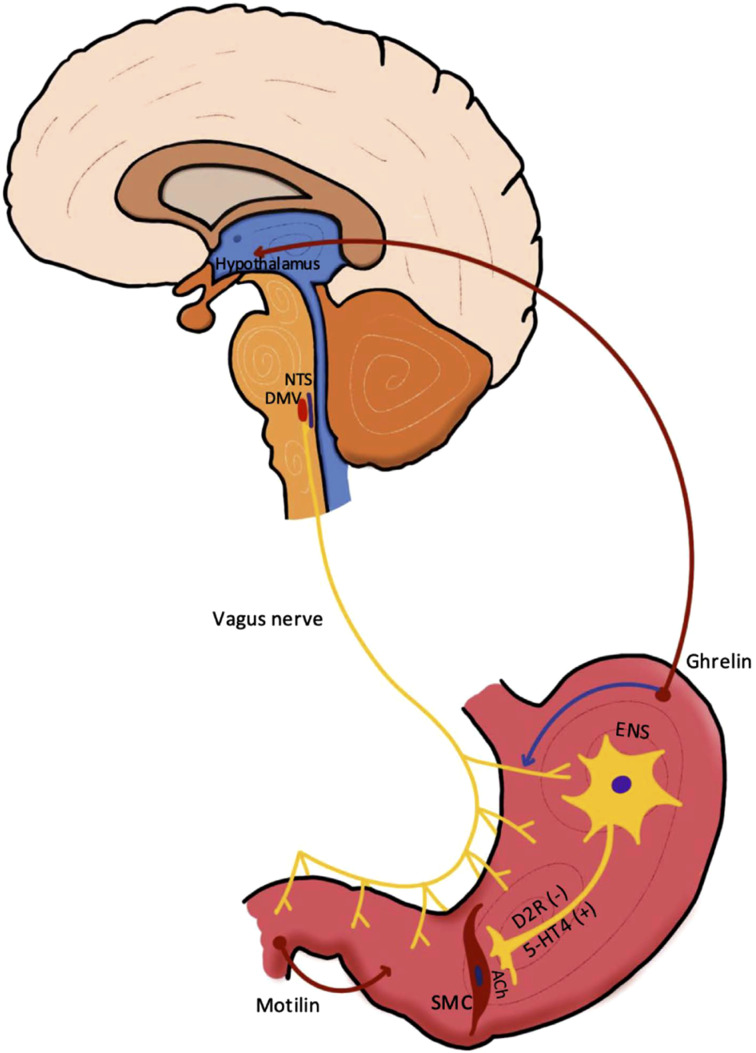
Regulation of Gastric Motility by Neurotransmitters and Hormones. Gastric Motility is Influenced by Both Neurotransmitters and Gut Hormones. Dopamine Reduces Motility by Inhibiting Acetylcholine Release Through D2 Receptors on Cholinergic Neurons, while Serotonin, via 5-HT4 Receptor Activation, Promotes Acetylcholine Release and Enhances Motility. Ghrelin, Known for its Role in Appetite Regulation, Affects Gastric Function by Acting on the Vagus Nerve, or Reaching the Hypothalamus Through the Bloodstream. Motilin, Secreted From the Duodenum, Increases Motility by Stimulating Cholinergic Activity in the Stomach’s Myenteric Plexus Through Blood Stream. Abbreviations: DMV, Dorsal Motor Nucleus of Vagus; NTS, Nucleus Tractus Solitarius; ENS, Enteric Nervous System; D2R, Dopamine D2 Receptor; 5-HT4, Serotonin 5-HT4 Receptor; ACh, Acetylcholine; SMC, Smooth Muscle Cell; Red Arrows Indicate Transmission of Gut Hormones via Blood Stream. (The Drawing in This figure was Created by the First Author, Dr Han-Lin Chiang.)

Medications used to treat gastroparesis act by modulating neurotransmitters in the enteric nervous system and regulating hormonal pathways to enhance gut motility (Figure 2). Presynaptic cholinergic neurons within the gastric wall release acetylcholine, which stimulates gastric smooth muscle contraction. The release of acetylcholine is inhibited by dopamine D2 receptors and facilitated by serotonin 5-HT4 receptors, both of which are located on presynaptic neurons. Therefore, D2 receptor antagonists and 5-HT4 receptor agonists enhance gastric emptying.^115^ On the other hand, ghrelin is a gut hormone released from endocrine cells in the oxyntic glands of the gastric fundus and corpus.^116^ It stimulates the vagus nerve and is also released into the bloodstream, where it acts on the area postrema and the hypothalamus to promote appetite and increase gastric emptying.^116^ Motilin, another gut hormone, is released from endocrine cells in the duodenum and jejunum. It enhances cholinergic activity within the myenteric plexus of the stomach, which in turn promotes gastric emptying.^116^ Finally, substance P, is a neuropeptide neurotransmitter released by the myenteric plexus neurons and act through NK1 receptor to enhance peristalsis and gut motility.^117^ Therefore, prokinetic agents with different mechanisms, including dopamine D_2_ receptor antagonists, motilin receptor agonists, serotonin 5-HT_4_ receptor agonists, cholinergic agents, NK-1 receptor antagonists, and ghrelin receptor agonists, may help treat gastroparesis. However, metoclopramide, a D_2_ antagonist that crosses the BBB, is the only FDA-approved medication for the treatment of gastroparesis; but is contraindicated in PD due to its potential to worsen motor symptoms. Studies on the effects of other prokinetics in patients with PD are limited. A summary of prokinetics that have been studied in PD is provided in Table 4.

**Table 4.** Summary of Prokinetics Agent That Have Been Studied in Patients With PD

Study	Drug	Study design	Number of patients	Outcome	Adverse effects
Dopamine D2 receptor antagonist					
Soykan 1997^118^	Domperidone 20 mg QID	OL, SA	11	Improved GI symptoms and GE time	Elevated prolactin: All nipple tenderness: 1
Serotonin 5-HT4 receptor agonist					
Asai 2005^119^	Mosapride	OL, SA	5	Reduce GE time and MF	None
Pinyopornpanish 2017^120^	Prucalopride 1-2 mg daily	OL, R, CO	10	Improve GE time compared with baseline and the domperidone group	Abnormal bowel sound: 3 Tiredness: 1 Abdominal pain: 1
Motilin receptor agonist					
Marrinan 2018^121^	Camicinal 50 mg QD	Phase II RCT	58	A trend of more rapid GE, not significant	Headache, fatigue (similar to controls), 2 nonfatal serious AE not related to camicinal (pruritus a)d circulatory collapse)
Histamine H2 receptor antagonist (act through noncompetitive inhibition of acetylcholine esterase)					
Doi 2014^122^	Nizatidine 300 mg/day	OL, SA	20	Significant shortening of GE time	None
Multi-action prokinetics					
Shin 2018^123^	DA-9701 30 mg TID	Phase IV RCT	38	Non-inferiority of DA-9701 vs domperidone	None
Choi 2020^124^	DA-9701 30 mg TID	Phase IV RCT	144	Improved overall GI symptoms and GI-related quality of life	Abdominal pain: 1 Constipation: 2 Nausea: 1 Sleep disturbance: 2 Dizziness: 2 Lethargy with slowness: 1 Skin rash: 1
Others					
Doi 2014^125^	Dietary herb Rikkunshi-to 15 g/day*	OL, SA	20	Significant shortening of gastric emptying (peak time of the (13)C-dose-excess curve)	None

*, 17% Atractylodis lanceae, 17% Poria cocos, 17% ginseng, 17% Pinellia ternata, 9% ginger, 9% orange peel, 9% Ziziphus jujuba, and 4% Glycyrrhiza; Abbreviations: OL, open label; SA, single-arm; R, Randomized; CO, cross-over; RCT, randomized controlled trial; GI, gastrointestinal tract; MF, motor fluctuation; GE, gastric emptying.

Domperidone is a peripherally acting D_2_ receptor antagonist and is considered safe for patients with PD as it does not increase the risk of motor deterioration. While not approved in the U.S. due to concerns over potential risk of sudden cardiac death, QT prolongation, and ventricular arrhythmias, it is approved in many other countries, including Canada, Australia, several European and Asian countries. Regulatory decisions vary by country based on risk-benefit assessments, with some imposing strict dosing limits (typically ≤30 mg/day) and additional precautions for patients over 60 and those with pre-existing cardiac conditions.^77^ The risk of QT prolongation is even higher when patients are prescribed other QT-prolonging medications, many of which are commonly used in patients with PD, such as apomorphine, quetiapine, certain tricyclic antidepressants, selective serotonin reuptake inhibitors, or serotonin-norepinephrine reuptake inhibitors.^126^ The gastrointestinal effects of DA-9701 in patients with PD have been investigated in 2 RCTs.^123,124^ DA-9701, a prokinetic agent derived from *Corydalis Tuber* and *Pharbitis Semen*, is approved in South Korea for treating functional dyspepsia. It acts as an agonist on 5-HT_1_A, 5-HT_4_, and α_2_ receptors, while antagonizing D_2_ receptors in the gastrointestinal tract.^127^ Shin et al conducted a randomized controlled trial in 38 PD patients, comparing DA-9701 to domperidone over 4 weeks. DA-9701 significantly improved MRI-based gastric emptying rates, while domperidone did not. Although levodopa plasma levels increased, the change was not statistically significant, and no worsening of motor symptoms was observed.^123^ In a larger placebo-controlled trial, Choi et al. examined DA-9701 in 144 PD patients with GI dysfunction.^124^ After 4 weeks, DA-9701 significantly improved GI-related quality of life scores, with continued benefits at 12 weeks in the open-label extension. The drug was well tolerated, and no significant impact on motor function was reported.^124^ These findings suggest that DA-9701 may enhance gastric motility and alleviate GI symptoms in PD without exacerbating motor symptoms.

## Constipation

### Epidemiology and Clinical Manifestation

Constipation is one of the most common non-motor symptoms in PD, not only affecting quality of life but also potentially leading to complications such as volvulus, megacolon, intestinal pseudo-obstruction, and bowel perforation.^128-132^ It can occur at any stage of the disease and may develop up to 20 years before the onset of motor symptoms.^133,134^ The reported prevalence ranges from 8% to 70%, largely due to variations in diagnostic criteria.^134^ Notably, a review of studies have indicated that the prevalence of constipation in the prodromal phase of PD ranged from 24% to 44%.^132,135^ Defining specific criteria for subjectively perceived constipation is difficult and various definitions have been employed. The Rome IV criteria is one of the widely used diagnostic criteria that include both colonic and anorectal symptoms in the evaluation of functional constipation.^136^ In the most updated Rome IV criteria, published in 2016, functional constipation is diagnosed when an individual experiences at least 2 of the following symptoms in at least 25% of defecations for at least 3 months, with symptom onset at least 6 months before diagnosis. These symptoms include straining, hard or lumpy stools, a sensation of incomplete evacuation or anorectal blockage, the need for manual assistance during defecation, and having fewer than 3 spontaneous bowel movements per week.^136^ Objectively, constipation is defined as slow colon transit time caused by decreased motility and reduced frequency of mass movements or presence of outlet obstruction due to lack of relaxation of the puborectalis muscle or anal sphincters during defecation.^137^

### Diagnosis

In most clinical practices, treatment for constipation typically begins after an initial history and physical examination, which may include a digital rectal exam to rule out secondary causes. However, in cases of suspected pelvic floor dysfunction with dyssynergic defecation or medication resistance, further objective evaluations are needed to refine treatment strategies (Table 3).^75^ If dyssynergic defecation, such as pelvic floor dyssynergia, rectal hyposensitivity with paradoxical contraction of the anal sphincter and puborectalis during defecation, or disorganized coordination of the abdominal muscles and diaphragm, are identified, treatment options including pelvic floor physical therapy,^138^ biofeedback therapy,^139,140^ or, in some cases, botulinum toxin injection into the puborectalis muscle or anal canal can be considered.^141,142^

### Pathophysiology and Treatment

Constipation related to slow colonic transit is the common cause in patients with PD.^143,144^ After excluding secondary causes, including medication use, lifestyle modification is suggested as the first step in treatment of constipation.^27^ Life style modification typically include increasing physical activity, fluid intake, and high-fiber diet.^145,146^ A structured daily routine can promote regular bowel movements by starting the morning with mild physical activity, a hot caffeinated beverage, and a fiber-rich cereal, all of which help stimulate the gastrocolic reflex, followed by responding to the first urge to defecate. In the evening, a fiber supplement (e.g. fiber tablet, psyllium) can further support stool consistency.^147^ In addition, asking patients to maintain an upright posture while seated on the toilet, limit their time to 5-10 mins to prevent hemorrhoidal congestion, use a footstool to adjust the anorectal angle, practice deep, relaxed breathing, and avoid straining may help facilitate better bowel movements.^146^ Furthermore, probiotics, usually classified as dietary supplements, have recently emerged as a useful option for alleviating constipation.^148^

Pharmacological therapy is usually considered a second-line treatment after lifestyle modification.^147^ Pharmacological treatments for constipation involve various pharmacological mechanisms, including stool softeners (e.g., docusate), osmotic laxatives (e.g., lactulose, polyethylene glycol [macrogol], magnesium based laxatives), stimulant laxatives (e.g., bisacodyl, senna, sodium picosulfate), prosecretory agents (e.g., lubiprostone, linaclotide), and prokinetics (5HT4 agonist, e.g., mosapride, prucalopride).^147^ Pharmacological treatment for constipation typically begins with osmotic laxatives, followed by stimulant laxatives if needed. When these are insufficient, prosecretory agents and prokinetics may be introduced. For immediate relief, rescue therapies such as glycerin suppositories, enemas with warm water or additional stimulant laxatives can be used.^27,145^ Importantly, there were previous misbeliefs that long-term use of stimulant laxatives could cause enteric neuronal damage, electrolyte imbalance, or tolerance. However, studies have shown that when used at the appropriate dose and frequency, stimulant laxatives do not cause enteric neuronal damage or electrolyte imbalance, and tolerance does not occur in the majority of patients.^149^ Dietary supplements and medications for constipation that have been evaluated in patients with PD are summarized in Table 5.

**Table 5.** Dietary Supplements and Medications for Constipation Symptoms of Patients With PD

Study	Drug	Study design (duration)	No. of pts with PD	Outcome	Adverse effects
Dietary supplements					
Astarloa 1992^150^	Fiber tablet (bran of wheat 375 mg, pectin 70 mg, dimethylpolyoxylhexane-900 2.5 mg)	OL, SA (2 mo)	19	BMs significantly increased (<2 to ≥ 4); higher bioavailability of levodopa	Flatulence:1
Ashraf 1997^151^	Psyllium 5.1 g/day	OL, SA (8 wks)	12	Increased stool frequency and weight but colonic transit or anorectal function no altered	None
Sakakibara 2005^152^	Dai-Kenchu-to 5 g/day TIDγ	OL, SA (12 wks)	6 PD 4 MSA	Significant improvement in total colon CTT, recto-sigmoid CTT, and rectal contraction (Videomanometry)	None
Osmotic laxatives					
Eichhorn 2001^153^	Mocrogol (13 g + electrolyte) 1 to 3 sachet	OL, SA (9 - 21 wks)	8 PD 2 MSA	Increased in the self-reported stool frequency (2-4/week), softening of stool, and increased ease of defecation	None
Zangaglia 2007^154^	Macrogol (7.3 g + electrolytes) 1 sachet BID	RCT (8 wks)	57	Responder rate significantly higher than the controls with significant increased in BMs (responder rate at week 8: 93.8% vs 46.7%) and stool consistency (responder rate at week8: 68.8% vs 20.0%)	NA
Prosecretory agents					
Ondo 2012^155^	Lubiprostone 24 μg BID	RCT (4 wks)	54	A marked or very marked clinical global improvement reported in 64% (treatment) vs 18.5% (placebo); significant improvement in CRS, VAS, and stools per day	Loose stool: 12 Persistent diarrhea: 1 Abdominal pain: 2 Stool discoloration: 1 Daytime sleepiness:1 Fatigue: 1 Rash: 1
Freitas 2018^156^	Linaclotide 145 or 290 μg/day	OL, SA (mean: 10 ± 8 mo)	16 PD 3 non-PD	BMs/week significantly increased. (1.5 to 3)	None
Serotonin 5HT4 agonist					
Jost 1993^157^	Cisapride 5 mg BID*	OL, SA (7 days)	20	Improvement of CTT	None
Jost 1997^158^	Cisapride 5 mg BID*	OL, SA (1 year)	25	The effect of CTT is most significant at 1 wk and reduce with time	no central side effects
Liu 2005^159^	Mosapride citrate	OL, SA (3 mo)	7 PD 7 MSA	13 out of 14 patients had improvements in bowel frequency and difficulty defecation; significant shortening of left colon and total colon CTT; lessened the first sensation of rectal filling & increased rectal contraction during defecation (Videomanometry)	No serious adverse effects
Sullivan 2006^160^	Tegaserod 6 mg BID**	RCT (4 wks)	15	Not significant in Subject’s global assessment of abdominal discomfort/pain, symptom relief, bowel habits, satisfaction with bowel habits, abdominal discomfort/pain, bloating, stool frequency, stool consistency, and straining	None
Freitas 2018^156^	Prucalopride 1-4 mg/day	OL, SA (19 ± 18 mo)	13 PD 4 non-PD parkinsonism	BMs/week significantly increased. (1 to 5)	Headache: 1
Probiotics					
Cassani 2011^161^	Fermented milk drink (65 mL) containing 6.5 × 109 CFU of *Lactobacilus casei* Shirota	OL (5 wks)	40	Significant improvement in the number of days per week of normal consistency of stool, feeling bloated, abdominal pain, and incomplete emptying	None
Barichella 2016^162^	Fermented milk, containing multiple probiotic strains and prebiotic fiber (125 g) QD	RCT (4 wks)	120	Significantly increased in the number of CBMs (mean increase: 1.2 (95% CI 0.8-1.6)); higher number of patients in the treatment group had ≥3 CBMs and increased by ≥ 1 CBS per week during week 3&4	Abdominal distention: 1
Ibrahim 2020^163^	Multistrain probiotics (Hexbio®)✣	RCT (8 wks)	48	Significant higher weekly BOF (BMs: 4.18 (treatment) vs 2.18 (placebo)); significant decreased in GTT (77.32 vs 113.54)	Abdominal bloating: 2 Dizziness: 2
Tan 2021^164^	Multistrain probiotics 1 capsule QDΩ	RCT (4 wks)	72	Significant improvement in the change in the average number of SBM per week during the last 2 weeks of intervention (1.0±2); significant improvement also seen in stool consistency, QoL related to constipation, and percentage of satisfied patients	Lethargy: 1
Du 2022^165^	Bacillus licheniformis and BIFICOψ	RCT (12 wks)	46	Significant increase in the average number of complete bowel movements per week (1.09±1.24), improved BSS, PAC-SYM, PAC-QOL, and degree of defecation effort score	None
Sun 2022^166^	Probio-M8 2 g/dayξ	RCT (3 mo)	82	Significant improvement in the PAC-QOL, number of CBMs (1.42 to 3.33)	None
Yang 2023^167^	Lacticaseibacillus paracasei strain Shirota (LcS)Φ	RCT (12 wks)	128	Significant improvement of constipation-related symptoms (Wexner score (−2.65 ± 2.30), BSS (0.42 ± 0.80), BMs (1.29 ± 1.80), PAC-QOL (−13.00 ± 11.15))	None
Andreozzi 2024^168^	Enterolactis Duoθ 1 sachet QID	OL, SA (12 wks)	30	Significant improvements in PAC-SYM score (1.4 to 0.9), number of CBMs (3.0 to 5.5) and BSS (2 to 3)	NA
Others					
Sakakibara 2015^169^	Nizatidine (selective histamine H2-receptor antagonist)150 mg BID	OL, SA (12 wks)	20	Significant decrease of the CTT in patients with slow CTT (n = 12)	None
Segal 2021^170^	Fecal microbiota transplant	OL, SA (24 wks)	6	Improvement in constipation Wexner score 24 weeks following FMT in 5 of 6 patients; improvement in frequency and consistency of the stool in all patients	Vasovagal pre-syncope: 1
The Parkinson study group 2017^171^	Remamorelin (ghrelin agonist)	RCT (14 days)	56	Recruitment goal not met	NA
Hauser 2019^172^	ENT-01 (squalamine phosphate)	OL, SA, phase 2a (ST2 steady dose for 4-6 days)	10 (ST 1) 34 (ST 2)	Over 80% of patients reached the primary efficacy endpoint (CBM/week: 1.2 to 3.6)	Nausea: 43% Loose stool: 43% Abdominal pain: 32% Flatulence: 6.8% Vomiting: 6.8% Worsening of acid reflux: 4.5% Worsening of hemorrhoid: 2.2%
Roy 2021^173^	STW5 (phytotherapeutic agent) 20 drops TIDδ	OL, SA (28 days)	44	An increased in stool frequency ≥3 BMs observed only in 4 patients (9%) – negative study	Worsening of constipation: 1
Camilleri 2022^174^	ENT-01 (squalamine phosphate)	RCT, phase 2b (up to 25 days)	150	Significant improvement in CBMs (0.7 to 3.2), SBMs, stool consistency, ease of passage	Nausea: 34.4% Diarrhea: 19.4% Vomiting: 8.6%
Vacca 2022^175^	PHGG (*Cyamopsis TetraGonolobus*) plus hyaluronate	OL, SA (6 wks)	34	Significant improvement in the PAC-SYM and CGI-S	Safe and tolerated
Hatano 2024^176^	Elobixibat 10 mg/day (ileal bile acid transporter inhibitor)	RCT (4 wks)	74	Between-group difference in SBM frequency was insignificant after adjusting for baseline and sex (primary end point). Significant improvement in the SBM at week 4 in the elobixibat group with improved scores of satisfactions and stigma	Abdominal pain: 1 Diarrhea: 11 Malabsorption: 1 Feces soft: 12

*, withdrawn from the market due to serious cardiac side effects of arrhythmias and QT interval prolongation; **,discontinuation by the manufacturer in the US with previous concerns about cardiovascular risks; Abbreviations: SBMs, spontaneous bowel movements; CBMs, complete bowel movements; QoL, quality of life; CFU, colony-forming unit; BSS, Bristol stool Scale; PAC-SYM, Patient Assessment of Constipation symptom; PAC-QOL, Patient assessment of constipation quality of life questionnaire; CGI-S, Clinical Global Impression Severity of Illness Scale; BMs, bowel movements; CTT, colon transit time; GTT, whole gut transit time; BOF, bowel opening frequency; CRS, constipation rating scale; VAS, visual analogue scale; OL, open-label; SA, single-arm; ST, stage; mo, months; wks, weeks.

γ, dietary herbal extract with 50% ginger, 30% “Nin-jin” [ginseng], and 20% “Sansho.

✣, 30 × 109 CFU, 2% fructo-oliogosaccharide, and lactose. The microbial composition of the probiotics were: *Lactobacillus acidophilus* (BCMC^®^ 12 130)107 mg, *Lactobacillus casei* (BCMC^®^ 12 313)107 mg, *Lactobacillus lactis* (BCMC^®^ 12 451)107 mg, (BCMC^®^ 02 290) −107 mg, *Bifidobacterium infantis* (BCMC^®^ 02 129)107 mg and *Bifidobacterium longum* (BCMC® 02 120)107 mg.

Ω, 10 billion CFU of 8 bacterial strains: L*actobacillus acidophilus, Lactobacillus gasseri, Lactobacillus reuteri, Lactobacillus rhamnosus, Bifidobacterium bifidum, Bifidobacterium longum, Enterococcus faecalis, Enterococcus faecium*.

ψ*Bacillus licheniformis*(2.5 × 109 CFU, 2 capsules each time, 3 times daily), *Lactobacillus acidophilus, Bifidobacterium longum, Enterococcus faecalis*(BIFICO,1.0 × 107 CFU per strain,4 capsules each time, twice daily.

ξ, *Bifidobacterium animalis* subsp. *Lactis* 3 × 1010 CFU/day.

Φ*Lacticaseibacillus paracasei* strain Shirota (LcS)-fermented milk, containing 1 × 1010 living LcS cells.

θ, Lacticaseibacillus paracasei DSM 34,154 and the prebiotic inulin, ≥ 8 billion CFUs/sachet.

δ, STW5 contains 9 herbal extracts (white iberide amara, angelica root, chamomile flowers, cumin, seed thistle, balm leaves, peppermint leaves, celandine and licorice root).

Some clinical trials have investigated constipation treatment in PD, but few large RCTs have been conducted. Zangaglia et al. conducted an 8-week RCT with 57 PD patients to assess the effect of macrogol on constipation.^154^ The primary endpoint, defined as complete symptom relief or significant improvement in stool frequency, straining, consistency, or use of rectal laxatives, showed a significantly higher responder rate in the macrogol group at weeks 4 (78.3% vs 25%) and 8 (80% vs 30.4%) (*P* = 0.0003 and *P* = 0.0012, respectively). Macrogol also increased mean stool frequency at both weeks 4 (5.7 ± 2.3 vs 3.4 ± 1.7; *P* < 0.002) and 8 (6.6 ± 2.7 vs 3.7 ± 1.9; *P* < 0.002) and improved stool consistency (*P* = 0.001 at week 4, *P* = 0.000 at week 8). There was no change in PD motor function (UPDRS Part III) or quality of life, suggesting that macrogol is well tolerated in PD patients.^154^ Another RCT by Ondo et al. evaluated the efficacy and tolerability of lubiprostone (48 μg/day), a chloride channel activator (prosecretory agent), in 54 patients with PD.^155^ After 4 weeks of treatment, there were significant improvements in the global impression of change (*P* = 0.001), the visual analog scale score for change in constipation (*P* = 0.001), constipation questionnaire scores (*P* < 0.05), and the number of stools per day (*P* = 0.001). Loose stool was the most common adverse effect, occurring in 48% of patients, but it was mild, self-limiting, and did not lead to study withdrawal.^155^

Several RCTs have demonstrated that various probiotics have been demonstrated to be effective in managing constipation in patients with PD (Table 5).^150-176^ A recent comprehensive review has provided valuable insights into this topic.^177^ In an open-label study of 40 PD patients, 6-week supplementation with fermented milk containing *Lactobacillus casei* Shirota (6.5 × 10^9^ CFU), alongside dietary therapy, significantly increased bowel movement frequency and improved constipation-related symptoms, including bloating, incomplete evacuation, and abdominal pain.^178^ Two double-blind, placebo-controlled randomized clinical trials have provided Class I evidence supporting probiotic use for constipation in PD.^179,180^ In the first trial (n = 120), patients received either fermented milk containing multiple probiotic strains (total 250 × 10^9^ CFU) with prebiotic fiber or placebo for 4 weeks. The probiotic group showed significant improvements in complete bowel movements, stool consistency, and reduced laxative use.^179^ In the second trial, a lower-dose multistrain probiotic capsule (10 × 10^9^ CFU) was given daily for 4 weeks. Compared to placebo, the treatment group (n = 34) experienced increased spontaneous bowel movements and improved stool quality and constipation-related quality of life.^180^ However, the potential benefits of probiotics beyond constipation remain unclear, and there is insufficient evidence to determine whether any specific probiotic formulation is superior to others. Given that the longest study duration to date is only 12 weeks, the long-term effects of probiotic use in PD are yet to be established. While existing studies indicate that probiotics do not cause serious adverse effects, caution is warranted in critically ill and immunocompromised patients.^177^ Based on the aforementioned studies, in the 2019 MDS Evidence-Based Medicine review update, polyethylene glycol (macrogol) and lubiprostone were classified as “likely efficacious” and “possibly useful”, while probiotics and prebiotic fiber were deemed “efficacious” and “clinically useful”.^181^ Notably, although the 5-HT4 agonists cisapride and tegaserod were among the first agents tested for treating constipation in PD, these medications have either been withdrawn from the market or discontinued in most countries due to serious cardiac adverse effects.

Medications with different mechanisms have been tested for the treatment of constipation in PD. ENT-01, also known as squalamine phosphate, acts by displacing alpha-synuclein electrostatically bound to nerve cell membranes and has been shown to prevent the aggregation of alpha-synuclein monomers both in vitro and in vivo.^182^ Additionally, it stimulates enteric neurons and improves GI motility in PD mouse models.^183^ Two Phase 2 studies of ENT-01 for constipation in PD have been published. The Phase 2a study was conducted in 2 stages to assess the pharmacokinetics and effects of ENT-01 on bowel function in PD.^172^ In Stage 1, 10 patients with severe constipation received escalating single doses, with an 80% response rate at 200 mg. Stage 2 involved 34 patients who received increasing daily doses until a prokinetic effect was observed or the maximum tolerated dose (250 mg/day) was reached. This was followed by a 7-day fixed-dose phase and a 2-week washout. Over 80% of patients met the primary efficacy endpoint, with complete spontaneous bowel movement (frequency increasing from 1.2 per week at baseline to 3.6 (*P* = 1.2 × 10^-7^), and spontaneous bowel movements rising from 2.6 to 4.4 per week. Stool consistency and ease of evacuation also showed significant improvement. However, these benefits diminished after washout, indicating the need for continued treatment. ENT-01 was well tolerated, with mild to moderate side effects such as nausea (47%), diarrhea (40%), and abdominal pain (32%). Systemic absorption remained below 0.3%, suggesting its effect is localized to the enteric nervous system.^172^ The Phase 2b RCT of ENT-01 enrolled 150 patients with PD and constipation.^174^ The treatment lasted for 25 days, starting with a dose-escalation phase until reaching the prokinetic dose or a maximum of 250 mg/day, followed by a washout period. The ENT-01 group showed a significant increase in the CSBM rate (from 0.7 to 3.2 per week) compared to the placebo group (from 0.7 to 1.2 per week; *P* < 0.001). Secondary outcomes, including SBMs (*P* = 0.002), stool consistency (*P* < 0.001), ease of passage (*P* = 0.006), and reduced laxative use (*P* = 0.041), also showed significant improvements. The most common side effects were nausea (34.4%) and diarrhea (19.4%), but the treatment was generally well tolerated.^174^

Elobixibat is an inhibitor of the ileal bile acid transporter (IBAT), which regulates bile acid reabsorption into the liver. By blocking IBAT, elobixibat increases bile acid levels in the intestinal lumen, promoting water and electrolyte secretion. Additionally, bile acids interact with transmembrane G protein-coupled receptors, leading to serotonin release, activation of enteric neurons, and stimulation of colonic motility.^184,185^ The efficacy of elobixibat for constipation has been demonstrated in a Phase 3 RCT.^186^ Consequently, Hatano et al investigated its effects in 74 patients with PD and constipation. Unfortunately, although there was a significant increase in SBM frequency from baseline to week 4 in the elobixibat treatment group (4.2 ± 2.6 to 5.9 ± 3.2), the study did not achieve its primary endpoint, as the between-group difference in frequency changes at week 4 was not statistically significant.^176^

### *Helicobacter* pylori and Small Intestinal Bacteria Overgrowth

The role of GI infections, particularly *Helicobacter pylori* (*H. pylori*) and SIBO, in PD has been extensively investigated, though findings remain conflicting.^74^ Nonetheless, these treatable infections are of clinical relevance, as they may contribute not only to GI discomfort but also to motor fluctuations in PD.^187,188^ Disruption of gastrointestinal integrity due to *H. pylori* infection can adversely affect levodopa pharmacokinetics and clinical response. *H. pylori* infection, especially when localized in the duodenum, may impair levodopa absorption through several mechanisms. First, *H. pylori*-induced gastroduodenitis leads to inflammation of the gastric and duodenal mucosa, disrupting normal absorptive function.^188^ Second, the infection alters gastric acid production, resulting in elevated gastric pH, which reduces levodopa solubility and hinders its uptake.^188^ Third, *H. pylori* may promote the generation of reactive oxygen species, which can damage levodopa molecules or interfere with their absorption.^189^ The resulting reduction in levodopa absorption can manifest clinically as worsened motor fluctuations, including more frequent and unpredictable “off” periods, diminished overall treatment efficacy.^190^

Several studies have shown that eradication of *H. pylori* via antibiotic therapy may restore gastrointestinal function and enhance levodopa absorption, thereby improving motor control and reducing fluctuations in PD patients. A systematic review and meta-analysis of 13 studies reported that PD patients with *H. pylori* infection exhibited more severe motor symptoms, including higher UPDRS motor scores, increased levodopa-equivalent dose requirements, prolonged time to “ON” state, and reduced “ON” duration.^191^ A randomized controlled trial involving 80 PD patients with confirmed *H. pylori* infection found that eradication have a trend to improve MDS-UPDRS motor scores at 12 weeks, although not reach the statistically significance level (*P* = 0.089), nor did it affect other motor or non-motor outcomes.^192^ These findings suggest that *H. pylori* infection can significantly compromise the effectiveness of levodopa therapy by altering the gastrointestinal environment. Early recognition and treatment of *H. pylori* infection should be considered in PD patients, particularly those with motor fluctuations or dyspeptic symptoms, to optimize therapeutic outcomes and overall quality of life.

SIBO causes symptoms including flatulence, bloating, chronic abdominal pain, diarrhea, steatorrhea, and malabsorption with weight loss and vitamin deficiency.^74^ It has also been implicated in affect levodopa absorption through inflamed or damaged intestinal mucosa. Fasano et al screened 33 patients with PD and 30 controls for SIBO and *H. pylori* infection. They found that patients with isolated SIBO had worse motor fluctuations, including longer off-time and more frequent episodes of delayed-on and dose failure. Treatment with rifaximin led to an improvement in motor fluctuations; however, 43% of patients experienced recurrence within 6 months.^193^ Another small open-label study included 14 patients with PD and levodopa-induced dyskinesia and motor fluctuations, treated them with sodium phosphate enema followed by oral rifaximin and polyethylene glycol for 7 and 10 days. Improvements in the UPDRS part IV, including duration and severity of dyskinesia, “Off” duration, functional impact and complexity of motor fluctuation were found.^194^ Conversely, another study screened 103 patients with PD and found 25.3% had SIBO. The SIBO-positive patients did not have worse motor fluctuation but was found to have worse score on “on” stage motor assessment (UPDRS part III).^195^ Due to inadequate evidence, the consensus published on behalf of the Other Non-Motor Features Working Group of the Parkinson Study Group recommended that SIBO evaluation should be reserved for patients with severe motor fluctuations and symptoms of malabsorption or maldigestion. Additionally, rifaximin is considered safe for use in patients with PD, while the decision to treat recurrent SIBO should be based on individual factors and clinical judgment.^20^

## Summary and Conclusion

**Figure 3.**
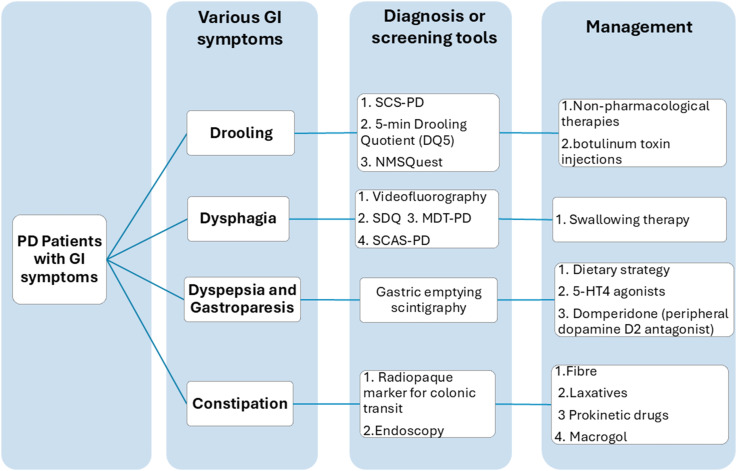
A Practical Algorithm for Evaluating and Management of Gastrointestinal Dysfunction in Patients With Parkinson’s Disease. (The Drawing in This figure was Created by the Corresponding Author, Dr Chin-Hsien Lin.)

The GI system plays a crucial role in PD, not only as a site of early pathological changes but also as a contributor to non-motor symptoms that significantly affect patients’ quality of life. While pharmacological and non-pharmacological approaches offer symptomatic relief, current treatment strategies are largely empirical and lack robust clinical evidence. Commonly used clinical approaches and therapeutic strategies are summarized in Figure 3, which illustrates a stepwise algorithm to guide proper assessment and management. By incorporating these clinical tools, the review is intended to translate current evidence into real-world practice and support clinicians in the day-to-day care of patients with PD experiencing gastrointestinal dysfunction.

Future research should focus on refining treatment algorithms, identifying biomarkers originated from the gastrointestinal tract for early diagnosis, and exploring novel personalized therapeutics to not only improve GI dysfunction in patients with PD but also mitigate disease progression. Future research should prioritize refining treatment algorithms, identifying reliable GI-derived biomarkers for early diagnosis, and developing personalized therapeutic interventions. In particular, emerging strategies aimed at restoring gut homeostasis, such as reducing enteric α-synuclein aggregation and enhancing intestinal motility, hold promise for not only alleviating GI dysfunction but also potentially modifying disease progression. These advances are critical to establishing precision medicine approaches for patients with PD.

## Data Availability

All data supporting the findings of this study are available from the corresponding author upon reasonable request. *
